# Environmental exposure and nanotoxicity of titanium dioxide nanoparticles in irrigation water with the flavonoid luteolin

**DOI:** 10.1039/d3ra01712e

**Published:** 2023-05-10

**Authors:** Epicurioua A. Frazier, Rajendra P. Patil, Chandrakant B. Mane, Daryoush Sanaei, Fahad Asiri, Seong S. Seo, Hamidreza Sharifan

**Affiliations:** a Department of Natural Sciences, Albany State University Albany GA USA Hamidreza.sharifan@asurams.edu; b Department of Chemistry, M. H. Shinde Mahavidyalaya Tisangi-416206 MH India; c Department of Chemistry, Shri Vijaysinha Yadav College of Arts and Science Peth Vadgaon MH India; d Center for Water Quality Research, Institute for Environmental Research, Tehran University of Medical Sciences Tehran Iran; e Environment & Life Sciences Research Center, Kuwait Institute for Scientific Research P.O. Box 24885 Safat 13109 Kuwait

## Abstract

Different concentrations of titanium oxide nanoparticles (TiO_2_NPs) have been frequently reported in treated wastewater used for the irrigation of crops. Luteolin is a susceptive anticancer flavonoid in many crops and rare medicinal plants that can be affected by exposure to TiO_2_NPs. This study investigates the potential transformation of pure luteolin in exposure to TiO_2_NP-containing water. In an *in vitro* system, three replicates of 5 mg L^−1^ of pure luteolin were exposed to TiO_2_NPs (0, 25, 50, 100 ppm). After 48 h exposure, the samples were extensively analyzed by Raman spectroscopy, ultraviolet-visible (UV-vis) spectroscopy, and dynamic light scattering (DLS). A positive correlation was found between TiO_2_NPs concentrations and the structural alteration of luteolin content, where over 20% of luteolin structure was allegedly altered in the presence of 100 ppm TiO_2_NPs. The increase of NPs diameter (∼70 nm) and dominant peaks in Raman spectra revealed that luteolin was adsorbed onto the TiO_2_NPs surface. Further, the second-order derivative analysis confirmed the transformation of luteolin upon exposure to TiO_2_NPs. This study provides fundamental insight into agricultural safety measures when exposed to air or water-borne TiO_2_NPs.

## Abstract 2

Luteolin is a susceptive anticancer flavonoid in many crops and rare medicinal plants that can be affected by exposure to TiO_2_ NPs in water systems.

## Graphical abstract

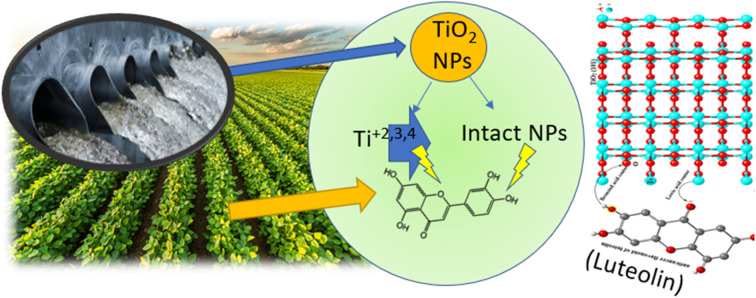

## Introduction

Irrigation of treated wastewater has become an increasingly popular solution for water scarcity and wastewater management worldwide.^1,2^ Treated wastewater is a valuable source of nutrients and water for agriculture, landscaping, and non-potable uses. However, using treated wastewater for plants and crop irrigation raises concerns about potential contamination with nanoparticles (NPs).^3,4^ NPs enter the wastewater treatment system through industrial discharges and agricultural activities.^5^ The presence of NPs in treated water pose risks to human health and the ecosystem. For example, the total dissociated Ti ions, the core ion of TiO_2_NPs, was reported in the range of 3200–43000 μg m^−3^ in treated wastewater.^1,6,7^ The built-up concentration of NPs and dissociated ions and bioaccumulation is of high concern.

According to the World Health Organization (WHO) report, about 80% of primary health care in many developing countries relies on medicinal plants.^8^ These plants contain secondary metabolites, known as Plant-Derived Medicinal Compounds (PDMC), which provide irreplaceable pharmacological properties for treating various diseases, including cancer. PDMC contains essential pharmacological properties with sustainable and low-cost biomass sources to be used as medicaments. Sustainable PDMC production is crucial for those who rely on nature-based products as well as the vegan population. However, environmental pollutants such as NPs affect plant growth, development, crop yield, and the metabolism of primary and secondary metabolites. The rapid development of nanotechnology has led to the high contamination of NPs, jeopardizing the production of high-quality and rare PDMC. Studies have shown that the PDMC contain reactive molecular groups (*i.e.*, OH) and surface charges that may trigger their potential reaction with NPs.^9,10^ Previous studies have also demonstrated that exposure to metal oxide NPs induces the release of antioxidant enzymes and changes the cellular macromolecule compositions in medicinal plants.^11,12^ It has been suggested that reactive oxygen species (ROS) which induces by metallic oxide NPs, also affect the transcription of secondary metabolites, leading to the alteration of PDMC.^13^ For example, TiO_2_ NPs upregulated anthocyanin and other flavonoid transcription in *A. thaliana*, *Oryza sativa* L and *N. tabacum*,^14–16^ and Ag NPs inhibited Ribulose-1,5-bisphosphate carboxylase/oxygenase (Rubisco) activity in *Spirodela polyrhiza* L.^17^ Despite the nanotoxicity effects, some studies have reported a positive response in the production of PDMC. For example, Fazel *et al.* 2016 reported that Ag–Au-NPs mixed with naphthalene acetic acid induced maximum level of medicinal flavonoid (6.71 mg g^−1^-DW) in *Prunella vulgaris* L.^18^ The effects of NPs on medicinal flavonoids in the literature is limited to soil and hydroponic studies.^19,20^ However, the airborne NPs contamination of medicinal plants and exposure to cellular PDMC is highly neglected.

Luteolin (5,7,3′,4′-tetrahydroxyflavone) is an anticancer PDMC in sensitive medicinal plants.^21,22^ Luteolin has been prescribed for a wide range of diseases due to its various medicinal properties.^23^ Its anticancer effects stem from its ability to inhibit tumor growth, induce apoptosis or programmed cell death in cancer cells, and modulate various signaling pathways commonly altered in cancer cells. Luteolin also exhibits anti-inflammatory and antioxidant activities, which may contribute to its anticancer effects by reducing oxidative stress and inflammation, two factors that can promote cancer development.

However, luteolin's aromatic structure and hydroxyl groups make it more sensitive to heavy metal and metal oxide NPs contamination. Studies have shown that luteolin acts as a metal chelator, which affects the bioavailability, original properties, and potential toxicity of various metals through the bioavailability of organic metal species.^24^ For example, divalent lead (Pb^2+^), which causes neurological and bone disorders, forms a chelate with luteolin.^21,25^ Furthermore, chelating of luteolin with divalent and trivalent cationic metals such as Ca^2+^, Zn^2+^, Mg^2+^, Fe^3+^, and Cu^2+^ have been frequently reported.^26,27^ Luteolin is highly susceptible to deformation due to poor hydrophobicity and bioavailability.^28^ However, the reaction of luteolin with intact NPs has not been investigated.

TiO_2_NPs in the atmosphere can agglomerate on plant surfaces and penetrate leaves through cuticle-free areas such as trichomes and/or stomata bases.^29^ However, their diffusion will be altered by the NPs' size, surface charge, concentration, and coating materials. Also, their interaction with secondary metabolites will vary depending on the active moiety of the reactive molecular groups. Despite many studies on the interaction of NPs with plants, understanding the dynamics of PDMC in response to NPs is still in its infancy. Recent investigations have shown the hormetic effects of TiO_2_NPs at a concentration range of 0–2500 mg L^−1^ on the growth, biochemical and physiological behaviors of the medicinal plant *Nigella arvensis*.^30^ For example, inhibitory effects were found at ≥1000 mg L^−1^ TiO_2_NPs, where chlorophyll and carotenoid synthesis were reduced.^30^ At 1000 mg L^−1^, TiO_2_NPs significantly promoted cellular H_2_O_2_ generation and increased antioxidant enzyme activities, including superoxide dismutase, ascorbate peroxidase, and catalase.^30^ Additionally, they enhanced total antioxidant content, total iridoid content, and 2,2-diphenyl-1-picrylhydrazyl scavenging activity. Therefore, the potential transformation of luteolin's structure and degradation due to direct exposure or indirectly through enzymatic and macromolecular alteration in response to TiO_2_NPs is highly expected.

TiO_2_NPs were found in both dissociated ion and intact NPs forms in cellular pH.^31^ However, their reciprocal effects on luteolin have not been investigated. This study investigated the potential interaction between luteolin and TiO_2_NPs-containing water in an *in vitro* system using extensive spectroscopy analysis. The specific objectives of this study were (i) to elucidate the effects of TiO_2_NPs on the transformation of luteolin under *in vitro* conditions and (ii) to provide evidence of the potentially destructive effects of TiO_2_NPs on luteolin. This investigation will provide a basis for further research on potential nanotoxicity levels of TiO_2_NPs on rare PDMC.

## Materials and methods

### Reagents

Negatively charged titanium oxide nanopowder (TiO_2_NPs) with the composition of anatase and 99% purity (20 nm) was purchased from US Research Nanomaterials, Inc. (Houston, TX). All reagents and solvents were provided at analytical grade quality. High-purity luteolin (≥98% (TLC), powder) was obtained from Sigma Aldrich (Milwaukee, WI). In each dispersion batch, deionized water (Millipore Milli-Q system, USA) was applied. All the laboratory glassware material was washed with soap and rinsed with 10% (v/v) HNO_3_ (Merck, Germany) before use.

### TiO_2_NPs Characterization

The TiO_2_NPs was extensively characterized before the exposure experiment by field emission-transmission electron microscope, FE-TEM (Hitachi 7700, Hitachi Ltd, Tokyo, Japan) and scanning electron microscope (JEOL JSM 6360). Briefly, a droplet of dispersed TiO_2_NPs in an aqueous phase was placed onto a carbon-coated copper grid, the grids were air-dried for 1 h until a thin layer of TiO_2_NPs was formed. The prepared sample was analyzed by TEM and SEM. The NPs size of all the samples was calculated by the controls method. The TiO_2_NPs structure was confirmed by X-ray diffraction using Philips PW-1710 X-ray diffractometer with CuKα radiation (*λ* = 1.54178 Å). The FT-IR spectra were recorded in the range of 400 to 1000 cm^−1^ on the instrument PerkinElmer, IR spectrophotometer (model E-2829) in KBr pellets.

### *In vitro* experiments

*In vitro* experiments in triplicate were used to study the TiO_2_NPs effects on the transformation of luteolin. TiO_2_NPs concentrations (0, 25, 50, and 100 mg L^−1^) were selected based on reported environmentally relevant TiO_2_NPs concentrations.^1^ The NPs dispersions of each *in vitro* bioreactor was prepared in 50 mL propylene centrifuge tubes. All the tubes, including the negative controls, were subjected to sonication at 25 °C with 150 W for 10 min to ensure full NPs dispersion. The pH of each solution was measured right after dispersion before the exposure process. The final concentration of 5 mg L^−1^ of luteolin was added to each tube. To evaluate the potential alteration of luteolin in short-term exposure, all the treatments were shaken at 100 strokes per min at a controlled temperature for 48 h at 25 °C (New Brunswick Scientific, Edison, NJ). After the luteolin–TiO_2_ NPs interaction process, the pH of the solution was measured. In parallel, three concentrations of TiO_2_NPs without luteolin (*n* = 3) and a series of pure luteolin without NPs were also prepared. Both intact NPs and luteolin were also subjected to the same sonication process. To filter the potential coagulates of luteolin with NPs and intact NPs from the luteolin species, 5 mL of the solution was centrifuged (8000×*g*) for 90 min using Amicon Ultra-15 centrifugal filter (NMWL = 3 kDa, Merck Millipore). The concentrations of luteolin in the filtrate were considered unreacted, or the byproduct was subtracted from the control containing only luteolin.

### Raman spectroscopy analysis

Raman spectroscopy was used to confirm the interaction of luteolin with the TiO_2_NPs and detect possible molecular alteration using a ProRaman-L (Enwave Optronics) with an excitation laser of *λ*_exc_ = 532 nm. The acquisition period was 10 s, and 15 average accumulations.

### Ultraviolet-visible (UV-vis) spectroscopy

The luteolin concentration in each treatment of the *in vitro* experiments was quantified using two methods of full scan method (200–700 nm) and the second-order derivative method of UV-vis spectroscopy 360. A sample of deionized water was scanned to subtract the baseline drift and noise before the sample was read. This experiment was processed under the condition of room temperature. The measurements were recorded at 2.5 nm absorbance mode for the sampling interval under fast scan speed (adjusting slit width to 5.0 nm). Eqn (1) shows the relationship for calculating the adsorbed luteolin onto TiO_2_NPs surface:1
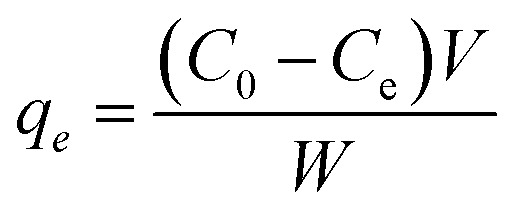
where *C*_0_ and *C*_e_ are the initial and final concentrations (mg L^−1^) of the luteolin, respectively, present in the equilibrium to analyze potential interaction. The adsorption capacity (*q*_e_) indicates the amount of luteolin (mg) adsorbed per unit of mass (g) of TiO_2_NPs, where *W* and *V* denote the mass of TiO_2_NPs and the volume of the batch solution (L), respectively.

### Nanoparticle size monitoring

To investigate the potential adsorption of luteolin on the surface of TiO_2_NPs, a solution containing only 25 ppm dispersed TiO_2_NPs and a solution containing a mixture of 25 ppm TiO_2_NPs and 5 mg L^−1^ luteolin after 48 hours (pH: 8) exposure were used. A volume of 2.5 mL from each solution was transferred to a 4.5 mL plastic cuvette with 10 mm optical path length (Fisher Scientific, USA) to measure the change of hydrodynamic size of TiO_2_NPs, using dynamic light scattering (DLS, N5 Submicron ParticleSize Analyzer, Beckman Coulter). Deionized water was used as the diluent with a scattering angle of 90°. The hydrodynamic particle size distribution for each sample was quantified through two 5 min sequences, and the reported values were the average of five continuous measurements. The surface charge was −39.2 ± 2.4.

### Structural analysis of luteolin–TiO_2_ NPs interaction

The molecular structural analysis for the TiO_2_ NPs was computed using Multiwfn software,^32^ which can describe the crystal structure of synthesized NPs. The interactions between the luteolin and TiO_2_ NPs were projected with VMD 1.9.4 visualization software.^33^

### Statistical analysis

Statistical analysis of luteolin concentrations after filtration was performed using Minitab 21.1 (Minitab Inc., State College, PA, USA). All datasets were acquired on a mean basis for triplicates in each treatment. The mean values of each experimental set were compared using a one-way analysis of variance (ANOVA) followed by Tukey's test. The significance level was 5% (*p* < 0.05).

## Results and discussion

### Characterization of TiO_2_ NPs

**Fig. 1:**
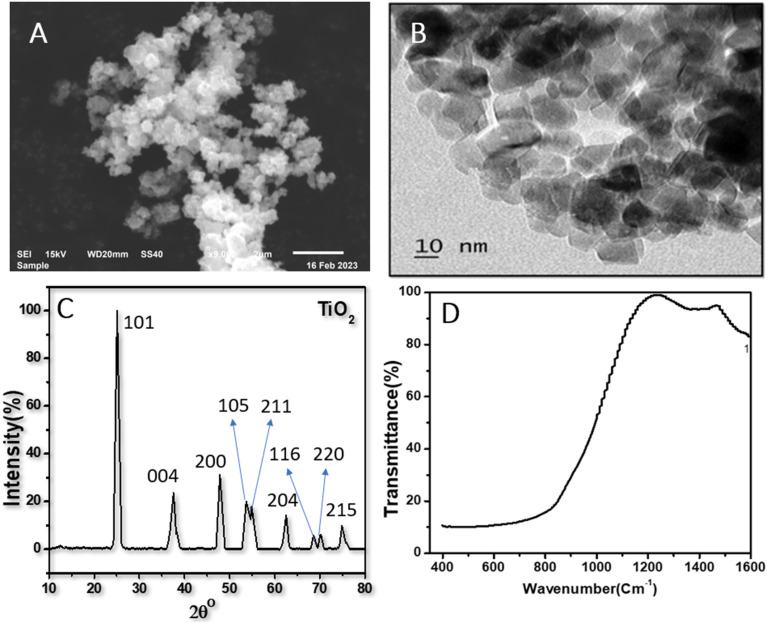
Charecterization information of the TiO_2_ nanoparticles (A) show a representative FE-SEM photograph, (B) TEM image, showing the homogenous size of dispersed NPs (C) XRD patterns of anatase phase and (D) FT-IR Spectra.

The representative FE-SEM and TEM images of the (101) facet of the TiO_2_ NPs sample are exhibited in Fig. 1A and B, indicating the octahedral morphologies. As shown in Fig. 1C, TiO_2_NPs have anatase properties with a well-crystallized structure, in good agreement with the JCPDS data file NO. 21-1272.^34,35^ The polymorph of anatase TiO_2_NPs has a dominant (101) facet (>98% of total area) owing to its high thermodynamic stability. In contrast, the other orientations were at minimal. According to the higher catalytic reactivity of other orientations than that of (101) surface, we found that TiO_2_NPs with dominant (101) facet could exhibit higher interaction with luteolin molecules due to semi-occupied surface area, reaching more exposure, resulting in high adsorption capacity. Fig. 1D shows the typical FT-IR spectra of TiO_2_NPs in the spectral region 400–1600 cm^−1^. The absorption bands observed within this range are an indication of the formation of single-phase metal oxides.

### Effects of TiO_2_NPs on the luteolin transformation using UV spectrum analysis

**Fig. 2:**
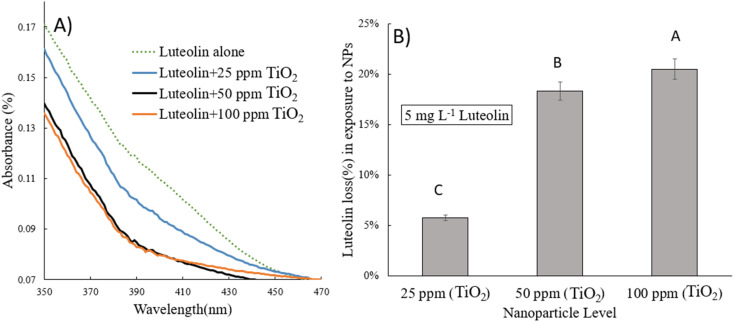
(A) UV-vis spectra of luteolin systems after 48 h exposure to three levels of TiO_2_NPs (25, 50 and 100 ppm) at pH 8 (A) and at pH 7.4 (B). Luteolin lost content % derived from the detected concentration of luteolin at 350 nm. Error bars signify standard deviation (*n* = 3). Different letters on top of each column indicate significant differences between luteolin lost in each treatment.

Typical UV-vis spectra of pure luteolin and its content in exposure to three levels of TiO_2_NPs are shown in Fig. 2A. Luteolin dominated the light absorption between 350–450 nm, with maximum absorption recorded around 350 nm, which was used to process the lost luteolin content (Fig. 2B). At three significant changes, exposure to TiO_2_NPs indicated a significant loss or transformation of the luteolin content. Exposure to 100 ppm TiO_2_NPs resulted in a maximum loss of 20%, and by reducing the TiO_2_NPs to half, the lost content decreased only by 18%, suggesting the threshold level of cellular damage may be less than 50 ppm TiO_2_NPs. Two primary mechanisms may explain luteolin's behavior in exposure to TiO_2_NP. The first pathway may be driven by the surface adsorption of luteolin onto TiO_2_NPs, as previous studies have shown the high potential of TiO_2_NPs on drug delivery and adsorption.^36^ However, the second mechanism suggests that luteolin may partially transform due to the destructive behavior of induced reactive oxygen species (ROS) in exposure to TiO_2_NPs. Therefore, further experimental analysis was carried out to elucidate the behavior of the luteolin in response to the toxicity of TiO_2_NPs.

**Fig. 3:**
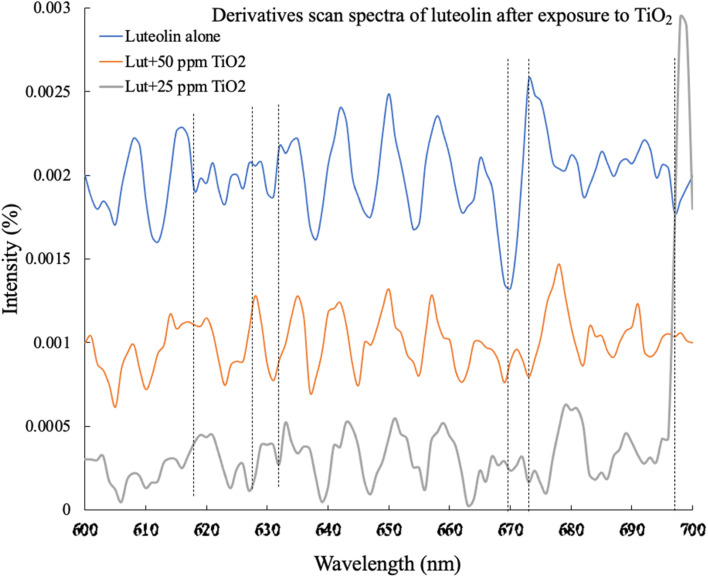
The second-order derivative UV-vis spectra of free luteolin and potential transformed molecular species or chelated luteolin–titanium(iv) after exposure to either 25 or 50 ppm TiO_2_NPs.

The intact and treated luteolin (second-order derivative) average spectra of UV-vis are presented in Fig. 3. The initial spectra (Fig. 2A) showed that the absorbance was mainly distributed in the 350–450 nm wavelength range. In contrast, the average spectra of the second-order derivative revealed that significant differences in absorbance occur at 618, 627, 632, 669, 673, and 697 nm, which can be assigned to the diversity of transformed molecules or byproducts according to their different structures. Results of the second-order derivative of UV-vis spectra preliminarily confirm the transformation of luteolin upon exposure to TiO_2_NPs. The bathochromic shifts that occurred at 618 and 669 nm may indicate the formation of a complex between luteolin and dissociated Ti^4+^ ions. Furthermore, significant bathochromic shifts have been observed in ligand-to-metal charge transitions when luteolin was exposed to free lead ions.^21^ According to the luteolin molecular structure, chelate formation with free metal ions could occur through two hydroxyl sites, 3′,4′-OH and 5,7-OH systems. However, the probability of chelating with free ions of the TiO_2_NPs is less due to the alkaline pH (>8) that prevents the dissociation of the NPs.

**Fig. 4:**
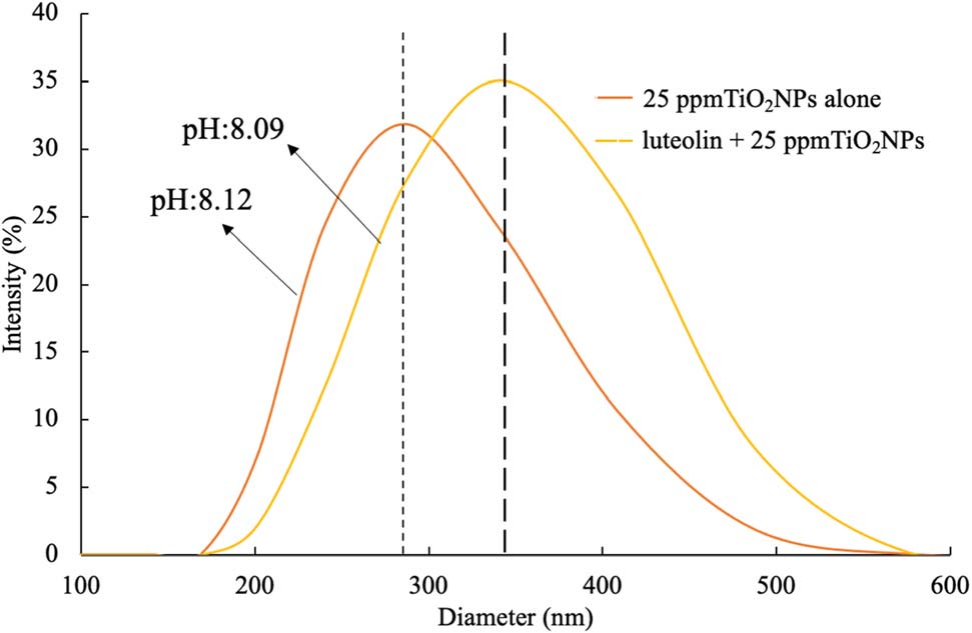
Alteration of the hydrodynamic sizes of TiO_2_NPs before and after 48 hours of exposure to 5 mg L^−1^ luteolin in DI water at pH 8. The plot represents the average of five scans.

The adsorption studies of organic materials onto the surface of TiO_2_NPs directly affect their hydrodynamic size. Fig. 4 illustrates the structural change in the diameter of TiO_2_NPs after exposure to 5 mg L^−1^ luteolin. The results show that at pH 8.1 ± 0.02, the hydrodynamic size of TiO_2_NPs significantly increased from a mean size of 290 nm to a larger size of 380 nm. This observation provides strong evidence of surface adsorption of luteolin after 48 h exposure to TiO_2_NPs. Previous studies have shown a similar approach to elucidate the response of plant's biomolecules in exposure to metallic oxide nanoparticles (*i.e.*, CeO_2_).^37^

### Effects of pH on the luteolin–TiO_2_ NPs interaction

**Fig. 5:**
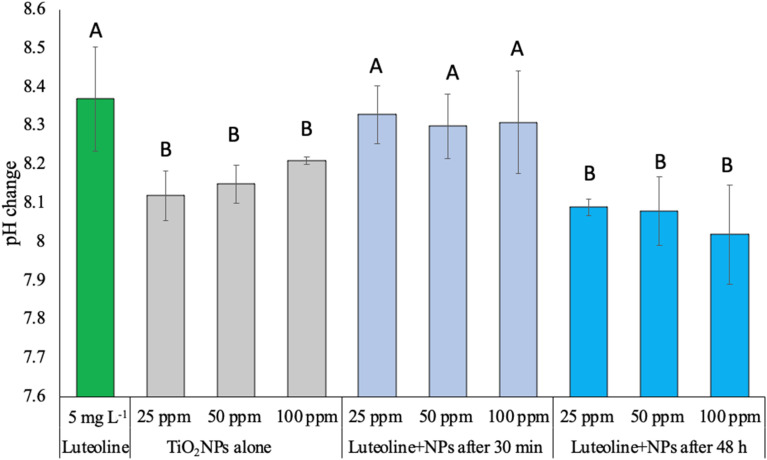
The trend of pH change at pure luteolin dispersion or its mixture with different concentrations TiO_2_NPs after and before adsorption. The letters above each data set represent statistical significance (*p* < 0.05). Error bars designate the standard deviation of each experiment (*n* = 3).

Fig. 5 displays the pH change of luteolin and three levels of TiO_2_NPs before and after exposure. The pure luteolin solution was slightly alkaline, with an average pH of 8.33. However, even after 30 minutes of exposure to three dispersed TiO_2_NPs levels, the luteolin solution's pH was still dominant, indicating that adsorption did not occur or only partially occurred. After 48 hours of interaction, the pH was significantly closer to the pH value that previously observed for intact TiO_2_NPs. However, no significant change was observed between different treatments, but the mean pH values slightly decreased with increasing concentrations of TiO_2_NPs. The reduction of pH may be explained by oxidative decarbonylation by reactive oxygen species (ROS), which promote the reduction of free H^+^ ions in the solution.^38^ A similar trend has been observed in the decarboxylation of mercaptobenzoic acid exposed to Ag NPs as a pH-dependent, where the shift of dissociated (R–COO–) to undissociated (R–COOH) took place until the equilibrium state.^39^ Further, Eshghi *et al.* 2023 suggested at lower pH values, the protonation of O–H groups of luteolin leading to higher molecular interactions,^40^ cause a repulsive electrostatic force between the luteolin molecules and attraction towards negative surface charge TiO_2_ NPs. This behaviour of luteolin was in a good agreement with pH studies of luteolin by Jurasekova *et al.* 2014.^41^ One common chemical modification of luteolin that can occur in alkaline solutions is called the “oxidative cleavage”,^41^ which typically results in the opening of the C-ring and the formation of two smaller fragments. This reaction is often catalyzed by metal ions, which can generate ROS that attack the flavonoid molecule.

### Structural analysis of luteolin–TiO_2_ NPs interaction

**Fig. 6:**
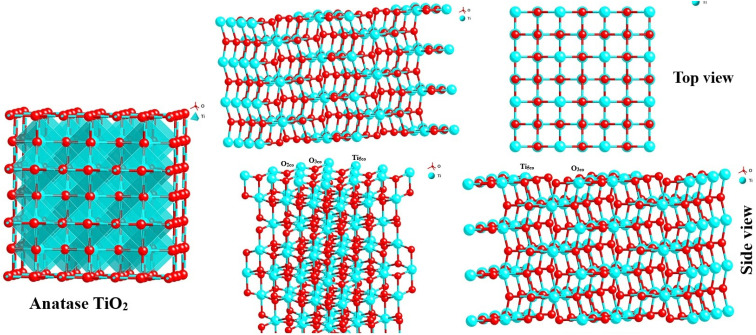
Three dimensional structure of bulk and surface anatase TiO_2_NPs (101) with different surface planes in both top and side views of structures.

According to the prepared anatase TiO_2_ NPs with the facet of (101) models (Fig. 6), the TiO_2_NPs surface atom (5 coordinated (Ti_5co_)) are coordinated to two O_2co_ atoms and two O_3c_ atoms of the top layer. In the (001) facet of TiO_2_ NPs, each Ti_5co_ atom in the surface and top layer is coordinated to one O_2co_ and three O_3co_ atoms, respectively. The Ti_6co_ sites are accessible and considered as main active sites only in the top layer of the (101) facet, whereas these sites exist in the underneath layer and bulk region. Due to the low coordination numbers of Ti_5co_ and O_2co_ sites in (101) than Ti_6co_ and O_3co_ sites in (001), the anatase TiO_2_NPs with (101) facet have more active and consequently more effective toward capturing luteolin.

On another side, by forming two OH groups surrounding the Ti^4+^ surface site in (101) facets formed through Ti–O–Ti bridges by water molecules, an H-bonding networks forms above every row of Ti^4+^ sites with luteolin on the surface-based reconstructed. These H-bond networks bridged between TiO_2_NPs and luteolin molecules are expected to effectively capture luteolin, consistent with the lower coordination numbers on the (101) facets. To explore the reactive sites further, Bader charge was carried out on the surface of TiO_2_. The estimated charges of the Ti_5co_ and O_2co_ active sites on the (101) and (001) surfaces are +1.58 and −0.88 e and +1.69 and −0.84 e, respectively. Notice the positive and negative values exhibit decreases and increases in valence electron numbers compared to neutral atoms, respectively.

Overall, the Ti_5co_ and O_2co_ sites on the anatase (101) facets of TiO_2_NPs can serve as Lewis acid and Brønsted base sites, respectively. As a result, the lone pair electrons of the O atoms in luteolin can interact with the Lewis acidic Ti_5co_ reactive sites, enhancing the luteolin capture capacity by TiO_2_NPs, as indicated by Raman spectra. Additionally, the O_2co_ sites can act as Brønsted bases and form hydroxyl groups by proton dissociation of the luteolin molecules, thereby promoting luteolin adsorption on the TiO_2_NPs surfaces. It is suggested that after luteolin adsorption, its OH groups bind to a Ti_5co_ site, and the hydrogen atom can then react with an adjacent O_2co_ atom to create a new hydroxyl group.^42,43^

### Raman spectral analysis of the luteolin–TiO_2_ NPs interaction

**Fig. 7:**
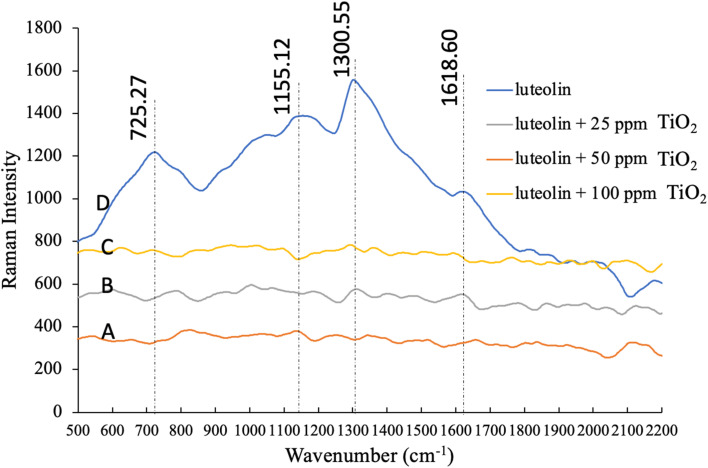
Raman spectra of pure luteolin in DI water and mixture of luteolin with dispersed 25, 50 and 100 ppm TiO_2_NPs after 48 hours. Four distinctive peaks at 725, 1133, 1300, and 1618 cm^−1^ are the prominent characteristic of pure luteolin in DI water.

To achieve a better understanding of the interaction between luteolin and TiO_2_NPs, we investigated both free luteolin and the mixed treatments with TiO_2_NPs by Raman spectroscopy. Raman studies that of the luteolin is often conducted in combination with metallic ions in organic solvents such as methanol,^44^ while we used the water as a natural aqueous phase. The pure luteolin Raman spectra in Fig. 7 showed four distinctive peaks at 725, 1133, 1300, and 1618 cm^−1^, which are prominent characteristic peaks. However, none of these peaks were detected when luteolin was exposed to TiO_2_NPs within 48 hours. In a study on the interaction of luteolin with Al(iii) ions, Rygula *et al.* 2013 suggested that Al ions were bonded from three connecting carbons to OH and a single carbon that double bonded to oxygen.^44^ However, we have not observed a potential dissociated Ti ions reaction with the luteolin. One possible interpretation is that the luteolin adsorbed onto the TiO_2_NPs' surface and settled, making it undetectable by Raman spectroscopy. Coagulation and formation of larger particles, previously confirmed by DLS analysis, also contributed to this effect. The associated spectra to either level of NPs were not significantly different, and they were shown at different intensities for better presentation. The Raman spectral analysis was consistent with the observation of a pH change, indicating the strong adsorption affinity of luteolin onto the TiO_2_NPs surface.

### Mechanisms to capture luteolin by TiO_2_ NPs

**Fig. 8:**
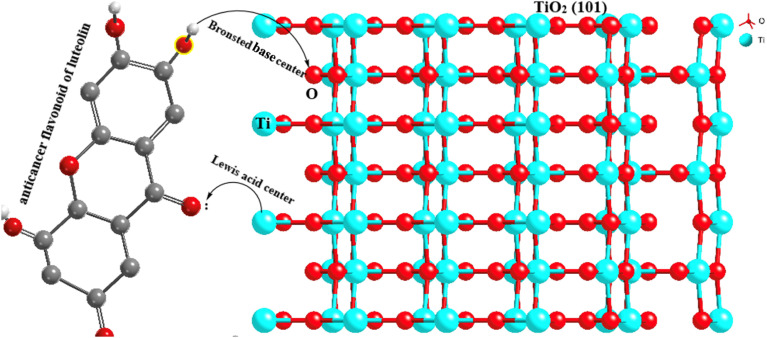
Exposure pathways of luteolin OH groups with the Ti^+^ (shown by the blue atoms) from the TiO_2_NPS surface on the first layer for anatase (101).

The effective capture of luteolin by TiO_2_NPs (101) can be attributed to the presence of both Brønsted and Lewis acidity. In Brønsted acidic sites, TiO_2_NPs (101) is capable of transferring a proton to the adsorbed luteolin molecule. This acidity is formed due to the tetrahedral coordination of Ti^4+^ or Ti^5+^ with oxygen.^45,46^ The nature of the adsorbed luteolin base could be one of the reasons for the effective capture of the Brønsted acidic center of TiO_2_ NPs (101). In the Lewis type of acidity, the TiO_2_NPs (101) plane surface accepts an electron pair from the adsorbed luteolin molecule, generating a coordinate bond. The octahedral Ti^+4^ and Ti^+5^ located on the edges of the (101) surface planes are the main Lewis centers that coordinate with luteolin.^47^ Also, synergistic interactions between the Lewis and Brønsted centers with luteolin molecules may occur.^48^ The schematic capture mechanisms of luteolin by TiO_2_NPs (101) surface planes are illustrated in Fig. 8.

## Conclusion

This study elucidated the potential transformation of luteolin anticancer flavonoids occurring at the interface of treated wastewater and anatase-TiO_2_NPs (101) with extensive spectrometric measurements. The possibility of breaking the luteolin to other molecules seems to be less based on the Raman spectra analysis and second-order derivative UV-vis spectra. However, the adsorption onto the anatase TiO_2_NPs (101) surface was confirmed with the DLS analysis. These results enhance the fundamental understanding of the transformation of Plant-Derived Medicinal Compounds in response to nanoparticle contamination. The results revealed the magnitude of impact is directly proportional to the level of nanoparticle contamination. Flavonoids can undergo chemical modifications in exposure to metallic oxide nanoparticles, but the specific type of modification that occurs may depend on various factors such as the pH, temperature, and the presence of other chemical species. However, it is worth noting that not all flavonoids are susceptible to this type of modification, and some may undergo other types of chemical reactions instead. Additionally, the specific conditions required for the reaction to occur may vary depending on the particular flavonoid and the intended application. Further studies are needed to investigate the impacts of crystal orientation on the molecular interaction of luteolin and other PDMC and challenging theirpersistency.

## Conflicts of interest

There is no conflicts to declare.

## Supplementary Material
